# Applicability of commercial clinical chemistry test kits for horse serum

**DOI:** 10.1186/s13104-020-05434-2

**Published:** 2021-01-07

**Authors:** Yoseph Cherinet Megerssa, Fikru Regassa Gari, Fanos Tadesse Woldemariyam

**Affiliations:** 1 Department of Biomedical Sciences, College of Veterinary Medicine and Agriculture, Addis Ababa University, Addis Ababa, Ethiopia; 2 Department of Biosystems, Division of Animal and Human Health Engineering, Laboratory of Host–Pathogen interaction, KU Leuven, Leuven, Belgium

**Keywords:** Clinical Chemistry, Horse, Validation

## Abstract

**Objective:**

Validation of a test method is critical for confirming that the test can generate accurate and precise data. Although commercial biochemical test kits exist there are no specific and validated commercial clinical chemistry test kits designed for horses. The aim of this study was to validate commercial clinical chemistry test kits designed for a human serum for use in horses.

**Results:**

Blood samples were collected from 29 apparently healthy adult male horses and pooled serum was prepared. Validation comprises replication and recovery experiments. Total observable error (TE_o_), sigma (σ) metrics, and quality goal index (QGI) were used to support the validation studies. Intra- and inter-assay variability was 2.05% and 2.08%, 2.26% and 1.89%, 2.4% and 1.63%, for total cholesterol, urea and total protein, respectively; recovery was 99.46%, 97.32%, and 100.1% for total cholesterol, urea and total protein, respectively. TE_o_% for the specified analytes was within the total allowable error (TE_a_). All three analytes satisfied the recommended requirement (> 3σ). The QGI for urea, as it had below 6σ was 0.95 indicating imprecision and inaccuracy. The results endorse the suitability of the studied commercial test kits and illustrated the acceptance criteria for horse’s serum.

## Introduction

Clinical laboratory plays a fundamental role in disease diagnosis, assessment of risk for a disease, monitoring to therapy and/or progression of the disease by providing timely data for patient management and disease surveillance [1]. One area in this regard is clinical chemistry laboratory and becomes popular in veterinary medicine [2]. It is indispensable that veterinary clinical laboratories must achieve accurate and precise test results. Clinical laboratory tests performed using an automated clinical chemistry instruments involves calibrators, controls, and reagents [3]. Ensuring the consistency of clinical chemistry laboratory test result is vital to maintain that testing is done right and produces accurate results [4].

Quality in health care has an immense impact on patient management as approximately 80% of all diagnosis is made on the basis of laboratory test results [5]. Method validation is one of the important quality mechanisms that are designed to ensure the generation of scientifically valid and useful analytical data [6]. Though all commercial clinical chemistry test kits are validated for their use in medical laboratories, they are also commonly used in veterinary clinical laboratories [7]. It is imperative to conduct partial validation studies, independent of the manufacturer’s claim. Partial validation should be made to confirm the analytical procedure is fit for its intended purpose to be eligible for use under actual settings [8]. According to the European Medicines Agency (EMA) guideline on bioanalytical method validation, commercial kits need to be revalidated to ensure that the sample analysis is performed accurately and precisely. Furthermore, a change of biological matrix or species is a reason to perform a partial validation, which can range from the determination of the within-run precision and accuracy to an almost full validation [9]. Validation is a pre-requisite to perform sample analysis and also key to satisfy regulatory requirements [10].

Clinical chemistry tests are often used for the measurement of analytes in serum and other body fluids. However, the quality of clinical chemistry tests may introduce systematic and random errors. This calls the need for validation of clinical laboratory tests, regardless of its use in diagnostic or research industry [11]. Therefore the aim of the study is to validate commercially available selected clinical chemistry in vitro diagnostic kits: urea, total protein, and total cholesterol designed for a human serum for use in horse serum. The research was the first partial validation study of clinical chemistry test kits in the veterinary clinical laboratory environment. Healthcare professionals and academia in Ethiopia and elsewhere will benefit at large from the findings.

## Main text

### Methods

#### Study design

The Study design was developed using the American Society of Veterinary Clinical Pathology (ASVCP) guidelines: allowable total error guidelines for biochemistry [11]. Total allowable error (TE_a_) for biochemical analytes was indicated in the guideline. Sample collection and animal use were approved by the institutional animal research ethics review committee at the Addis Ababa university, College of veterinary medicine and agriculture (Certificate reference no VM/ERC/09/01/12/2020).

#### Study animals and sampling technique

The World Organization for Animal Health (OIE) guideline 3.6.6 selection and use of reference samples and panels recommended minimum of 5 samples to prepare serum pool [12]. In addition to computing a statistically valid number of samples as suggested by Bayes Success-Run Theorem for validation studies 95% confidence and 90% reliability used. Therefore n = 28.4. We used 29 samples for the study [13]. The study animals were an adult male horse of age (≥ 4.5 years) and whose body condition score 3 were recruited by convenient sampling technique at society for the protection of animals abroad (SPANA) Ethiopia clinic. Apparently healthy horses from owners who were consent after being informed about the purpose of the study were physically examined. Horses with a history of medication excluded due to the possible impact of drugs on analysis.

#### Blood collection and processing

Blood samples from study animals were collected by a veterinarian from the jugular vein using the standard operating procedure. The blood was allowed to clot at room temperature for between 30 min and serum was separated from the red blood cells by centrifugation at 1200×*g* for 10 min at 4 °C. Serum was immediately transferred to polypropylene tubes (Eppendorf Safe-Lock tubes) and stored at − 20 °C until measurements. Samples were collected during two weeks in January 2020. Pooled serum samples were created by mixing equal volumes of individual serum then homogenized using an agitator for 10 min at 180 rpm. After homogenization aliquots of the homogeneous pool were divided into twenty portions to avoid the effect of repeated thawing and freezing.

#### Analytical validation

To examine the accuracy and precision of commercial clinical chemistry kits (JOURILABS diagnostics reagents and stains-Addis Ababa, Ethiopia) total cholesterol, urea, and total protein were used and analysis of the parameters was determined by the methods/techniques described as follows: urea by kinetic urease/GLDH (Glutamate dehydrogenase), total protein by biuret and total cholesterol by CHOD-PAP (cholesterol peroxidase4-aminophenazone). The procedure of validation was adopted from the Westgard JO method validation protocol. The analytical validation comprises recovery studies for accuracy and replication experiments for precision [14]. The tests were performed on semi-automated chemistry analyzer (EMP-168 biochemical analyzer Chengdu Empsun Medical Technology Co., Ltd. China).

#### Replication experiments

Precision was assessed by evaluating the intra- and inter-assay variability using the pooled serum. Intra-assay variability (repeatability) was determined by measuring total cholesterol, urea, and total protein in the same sample 20 times sequentially within a single run. Inter-assay variability (reproducibility) was determined by analysing the same sample in duplicate for 20 consecutive days. To avoid the effect of repeated thawing and freezing, the samples used for the determination of inter-assay were aliquot and stored at − 20 °C until use [15].

#### Recovery experiments

The spike and recovery (SAR) assessment is essential for the analysis and accuracy evaluation of the method for particular sample types. Spike and recovery assay is used to determine whether the detection of an analyte is affected by biological sample matrix and differences in the standard curve diluent [16, 17]. Serum samples were spiked with different concentrations of standard Total cholesterol (26 mg/dl; 0.1 ml of 200 mg/dl standard solution was spiked in 1 ml serum) Urea (9.1 mg/dl; 0.1 ml of 100 mg/dl standard solution was spiked in 1 ml serum) and total protein (1.1 mg/dl; 0.1 ml of 12 mg/dl standard solution was spiked in 1 ml serum).

### Quality requirement

#### Total allowable error (TE_a_)

The performance of tests was assessed by computing TE_obs_ (%) and σ values. TE_obs_ (%) = 2 × CV + bias (%), Bias (%) = [(target − measured) ÷ target] × 100%, where “target” is the spiked value for analyte and “measured” is the measured analyte concentration. The CV and bias (%) values from inter-assay were used to calculate TE_obs_ (%). If TE_obs_ (%) is less than TE_a_ (%); the quality requirement passes and no further action needed. The TE_a_ (%) employed in this study was total cholesterol: 20%, Urea: 12% and total protein: 10% adopted from the American society of veterinary clinical pathology (ASVCP) guidelines: allowable total error guidelines for biochemistry [11].

#### Sigma metrics (σ)

Sigma value (σ) calculated as σ = [TE_a_ (%) − Bias (%)] ÷ CV. The interpretations of the σ values are > 2: Poor, > 3: Marginal, > 4: Good, > 5: Excellent, and > 6: World-class. Acceptable performance of a method is declared if TE_obs_ < TE_a_ [18, 19].

#### Quality goal index ratio (QGI)

QGI describes the extent to which both precision and bias meet their respective quality. This is used to find the reasons for the lower σ in analytes whether the problem is due to imprecision or inaccuracy or both. QGI ratio calculated as QGI = Bias/1.5 × CV %. The interpretations of the QGI with low σ values (< 6) are QGI < 0.8 shows imprecision, QGI 0.8–1.2 shows both imprecision and inaccuracy and QGI > 1.2 depicts inaccuracy [20].

#### Data analysis

Statistical analyses were performed using IBM SPSS 20. The normality distribution of the data was tested using the Kolmogorov–Smirnov test prior to statistical analysis. Data of accuracy from bias and precision from intra-assay and inter-assay CVs were estimated using routine descriptive statistical procedures.

## Results

The present study validated total cholesterol, urea, and total protein test kits using pooled serum collected from 29 apparently healthy horses. Intra-assay precision was done by repeated measurements of pooled serum under specific and identical conditions on the same day. For inter-assay repeatability, the pooled serum was frozen in separate vials at − 20 °C, thawed at room temperature, and assayed on 20 consecutive days. The data generated was calculated in terms of mean SD and CV is presented in Table 1.

**Table 1:** Precision of the pooled serum for total cholesterol, urea and total protein

Precision	Intra-assay (N = 20)			Inter-assay (N = 20)		
Parameters	Mean	SD	CV	Mean	SD	CV
Total cholesterol	80.3	1.65	2.05	80.4	1.67	2.08
Urea	80.3	1.82	2.26	80.4	1.52	1.89
Total protein	6.25	0.15	2.4	6.13	0.1	1.63

To assess accuracy, a recovery method based on standard addition was used to evaluate the ability of the assay to recover the amount of analyte added to baseline pooled serum. The baseline pooled serum was obtained by the dilution of pooled serum with distilled water. While the spiking was done by the addition of standard solutions to pooled serum then both diluted and spiked pooled serum was assayed on 5 replicates and the average value is depicted in Table 2.

**Table 2:** Recovery for the pooled serum for total cholesterol, urea and total protein

Analyte	Addition	Dilution	Observed	Expected	Recovery (%)
Total cholesterol	93.6	75.5	18.1	18.2	99.46
Urea	66.72	64.7	8.86	9.1	97.32
Total protein	7.49	6.38	1.11	1.1	100.1

The TE_o_ was expresses by combining random error (% CV) from the precision and systematic error (bias) from the accuracy estimation. The TE_o_ for the specified analytes was within the TE_a_ indicated in ASVCP guidelines (Table 3). The quality of testing also assessed by sigma metrics and all analytes satisfied the recommended requirement (> 3 sigma values). Total cholesterol and total protein showed > 6σ zone (world-class quality) while urea showed 4.9σ (Good class quality). The QGI for urea, as it had below 6σ was 0.95 falling in the range of 0.8–1.2 shows both imprecision and inaccuracy (Table 3).

**Table 3:** The sigma metrics and quality goal index ratio for Total cholesterol, Urea, and Total Protein

Analyte	Bias (%)	CV (%)	TE_o_ (%)	TE_a_ (%)	Sigma	QGI	Problem
Total cholesterol	0.54	2.08	4.7	20	9.34	0.17	None
Urea	2.68	1.89	6.46	12	4.9	0.95	Impression and inaccuracy
Total protein	0.1	1.63	3.36	10	6.1	1.37	None

## Discussion

This study was undertaken to determine whether commercial clinical chemistry test kits are applicable to test analytes in horse serum. There are few studies on the validation of commercial kits and this study is the first in a veterinary laboratory environment in Ethiopia. Our study focused on the recovery and repeatability experiments which then followed by calculating sigma values and quality goal index for three analytes namely total cholesterol, urea, and total protein in horse serum.

According to the findings of the study for the intra-assay and inter-assay precision to be accepted, SD must not exceed 0.25 × TE_a_ and 0.33 × TE_a_ respectively for the given analyte [15, 21]. In this regard intra-assay and inter-assay precision for total cholesterol demonstrated < 5% and < 6.6%, urea < 3% and 3.96%, total protein < 2.5% and < 3.3%. The precision profile representing the %CV is within the established acceptance criteria.

The findings of recovery percentages were between expected values and measured values demonstrate that all tests were within the acceptance range of 80–120% [22, 23]. Besides the error observed was less than the allowable error assigned for the analytes [11]. Quality index ratios for total cholesterol and protein indicate no problem in terms of accuracy and precision  while in case of urea the root cause for impression and inaccuracy should be investigated before it routinely used as the quality of the test in such cases cannot be assured [24, 25].

## Conclusion

Validation of the bioanalytical methods should be an integral part in laboratory management and health care. Commercial clinical chemistry test kits are often validated by the manufacturers. There is a need to verify the validity of the test kits before applying to diagnostic and research purposes particularly when the sample matrix is different. The study demonstrated that the commercial kits used in the study satisfied the acceptable criteria and recommended its use for horse serum. However, a full validation study of the clinical chemistry test kits for their fitness in a number of laboratories and clinical decision limit is recommended.

## Limitations

- The study was unable to conduct comparison studies due to financial constraints.
- The study was unable to conduct validation on high and low concentration due to the unavailability of materials.
- The study was limited to conduct on male horse.

## Data Availability

The data used to support this study are available from the corresponding author on request.

## Abbreviations

AAU: Addis Ababa University
ASVCP: American Society of Veterinary Clinical Pathology
CV: Coefficient of variation
EMA: European Medicines Agency
OIE: World Organisation for Animal Health
QGI: Quality goal index
SAR: Spike and recovery
SD: Standard deviation
SPANA: Society for Protection of Animal’s Abroad
TE_a_: Total allowable error
TE_o_: Total observed error
